# Erythropoietin Receptor/β Common Receptor: A Shining Light on Acute Kidney Injury Induced by Ischemia-Reperfusion

**DOI:** 10.3389/fimmu.2021.697796

**Published:** 2021-06-30

**Authors:** Yuanyuan Wu, Bin Yang

**Affiliations:** ^1^ Basic Medical Research Centre, Medical School, Nantong University, Nantong, China; ^2^ Nantong-Leicester Joint Institute of Kidney Science, Nephrology, Affiliated Hospital of Nantong University, Nantong, China; ^3^ Department of Cardiovascular Sciences, College of Life Sciences, University of Leicester, Leicester, United Kingdom

**Keywords:** acute kidney injury, apoptosis, EPOR/βcR, fibrosis, inflammation, ischemia-reperfusion

## Abstract

Acute kidney injury (AKI) is a health problem worldwide, but there is a lack of early diagnostic biomarkers and target-specific treatments. Ischemia-reperfusion (IR), a major cause of AKI, not only induces kidney injury, but also stimulates the self-defense system including innate immune responses to limit injury. One of these responses is the production of erythropoietin (EPO) by adjacent normal tissue, which is simultaneously triggered, but behind the action of its receptors, either by the homodimer EPO receptor (EPOR)_2_ mainly involved in erythropoiesis or the heterodimer EPOR/β common receptor (EPOR/βcR) which has a broad range of biological protections. EPOR/βcR is expressed in several cell types including tubular epithelial cells at low levels or absent in normal kidneys, but is swiftly upregulated by hypoxia and inflammation and also translocated to cellular membrane post IR. EPOR/βcR mediates anti-apoptosis, anti-inflammation, pro-regeneration, and remodeling *via* the PI3K/Akt, STAT3, and MAPK signaling pathways in AKI. However, the precise roles of EPOR/βcR in the pathogenesis and progression of AKI have not been well defined, and its potential as an earlier biomarker for AKI diagnosis and monitoring repair or chronic progression requires further investigation. Here, we review biological functions and mechanistic signaling pathways of EPOR/βcR in AKI, and discuss its potential clinical applications as a biomarker for effective diagnosis and predicting prognosis, as well as directing cell target drug delivery.

## Introduction

Acute kidney injury (AKI) is a critical syndrome characterized by a sudden decline of renal function, with high morbidity and mortality (1–3). The poor prognosis of AKI is also evidenced by the high risk of progression to chronic kidney disease and end-stage renal disease over time, characterized by tubulointerstitial fibrosis (4, 5). Effective management of AKI is urgently needed including early diagnosis as well as target-specific treatment.

Renal ischemia-reperfusion (IR) injury is a common contributor to AKI, which could result from various clinical settings such as kidney transplantation, cardiac surgery, shock, vomiting, diarrhea, and burns (6). Parallel to injury, the kidney also develops defensive responses to restrict cellular damage and promote repair, one of which is associated with the classical hormone erythropoietin (EPO). Recombinant EPO, 5000 U/L in hemoperfusate, markedly decreased apoptotic cells in tubular areas, but increased the apoptotic cell death of inflammatory cells and their clearance in isolated porcine kidneys (n = 6) (7). *In vivo*, EPO at 1000 U/kg significantly improved renal function and structure at the acute stage (24 h) of IR-induced AKI in rats (n = 6) (8). These observations suggest a therapeutic opportunity of using EPO to improve the outcome of AKI. However, a large dose of EPO is needed for tissue protection, so it often results in side effects such as hypertension and thrombosis due to its high affinity to the classical homodimer (EPOR)_2_. The renoprotective receptor of EPO was a complex composed of the EPO receptor and β common receptor (EPOR/βcR), also known as innate repair receptor (9), but EPO has a low affinity to EPOR/βcR. Therefore, EPO-derived helix B surface peptide (HBSP) or its cyclic form CHBP was designed, which recognizes only EPOR/βcR, but not (EPOR)_2,_ thus dissociating tissue protection and erythropoiesis (10).

Our previous work demonstrated the renoprotective effects of HBSP and CHBP in a series study using both animal and cellular models. It was shown that the expression of EPOR/βcR was greatly triggered by IR injury in kidneys, and in particular located in tubular epithelial cells (TECs) (11), while its ligand HBSP or CHBP protected the kidney against IR-induced AKI, as well as cyclosporine A-induced fibrotic damage in kidneys (12). Mechanically, HBSP or CHBP was found to regulate the activation of transient receptor potential melastatin 7 ion channels and endoplasmic reticulum stress in tubular epithelia (13, 14). Exploring the role of EPOR/βcR in renal IR-induced AKI could help researchers to better understand its self-defense mechanisms, its signaling pathways and associated outcomes, as well as assessing its role as a potential biomarker to facilitate timely diagnosis, monitor the progression of kidney injury, and direct cell target drug delivery.

## EPO and its Derivatives

EPO is a highly-glycosylated protein with a molecular weight of around 30.4 kDa (15). In adults, interstitial fibroblasts in the kidneys are the major cells of EPO production, which maintain the level of EPO in the circulation by feedback signals (16). The main function of EPO is enabling the terminal differentiation of erythroid progenitor cells by inhibiting apoptosis and activating pro-survival signaling pathways (17–19). However, the action of EPO is not restricted to the hematopoietic system. Initial research observed a neuroprotective effect of recombinant EPO against ischemia-induced neuron damage in the brain (20). A similar effect was also described in other organs including the kidney, heart, and liver against ischemia-related injuries, by improving autophagy, anti-apoptosis, and anti-inflammation (21–23). It indicates that these organs have a receptor of EPO, which mediates the protection of tissues. Accumulated evidence suggests that heterodimer EPOR/βcR is responsible for the tissue protective effects (24–26). Upon injury, the expression of EPOR/βcR is instantly elevated, but EPO production in the site of injury is usually postponed, for example in astrocytes of the brain (27). Thus, there is a therapeutic window for exogenous supplement of EPO and its derivatives (9). Due to the much lower binding affinity of EPO to (1-20 nM) EPOR/βcR than to classic homodimer (EPOR)_2_ (100-200 pM) (28), a significantly higher dose of EPO is required to induce cytoprotection compared to erythropoiesis. Nevertheless, a high dose of EPO showed no significant tissue-protective effect in clinical trials, including four trails of renal transplantation (29–32) and one trail of AKI (33). Additionally, a number of studies indicated a high risk of mortality and adverse events related to the cardiovascular system by raising hematocrit and enhancing the activation of platelets and endothelia after EPO administration (34–37). Therefore, the application of EPO is restricted due to significant risk compromising its benefit. Looking into the 3D structure of EPO, helices A, C, and D binding with (EPOR)_2_ are associated with erythropoiesis, but only aqueous helix B binding with EPOR/βcR is related to tissue protection. Therefore, EPO was modified in a variety of ways to generate molecules retaining tissue protection and avoiding hematopoiesis, which included desialated EPO, carbamylated EPO (CEPO), and glutaraldehyde EPO. However, these EPO derivatives have different pitfalls including either short half-life, immune stimulation, or tissue permeability and compatibility (38–42).

Nevertheless, a small peptide derived from EPO, helix B surface peptide (HBSP), was designed and synthesized. It is a linear peptide formed by 11 amino acids, derived from the exterior aqueous surface of helix B, also known as ARA290 (10). HBSP possesses specific and powerful roles in tissue protection but without the side effect of erythropoiesis (43). Unfortunately, the plasma half-life of HBSP is short, around 2 min, which might affect its function *in vivo*. Scientists from the Chinese Academy of Sciences optimized the metabolic stabilization of HBSP in plasma by the conformational constraining of the head-to-tail connection that forms cyclic HBSP (CHBP) *via* thioether. CHBP gained proteolytic resistance significantly and prolonged its half-life time to at least 300 min *in vivo* (44). Preclinical studies proved that CHBP was renoprotective against IR injury in rodents (45) and large animal porcine models (3). The clinical application of CHBP, in particular HBSP, is promising as it is easily obtained, is an ambient dry powder for transportation, has low cost, and potential high efficacy for organ protection (45). However, metabolic characteristics such as serum stability, safety, tissue permeability, and cytoprotective efficacy in humans still need to be further investigated.

The effectiveness of a ligand drug is not only dependent on its specificity and tissue permeability, but also relies on the responsiveness of the receptor type. EPOR was reported to be associated with chronic fibrosis of the kidney by receiving persistent signals from EPO or excessive expression (46, 47). It was also reported that recombinant EPO provided renoprotection against fibrosis in adenine-induced CKD (48). However, EPO protected the IR kidney at the acute stage but promoted renal fibrosis in the long term. It is necessary to note that recombinant human EPO greatly increased myofibroblast numbers and collagen deposition in kidneys at 28 days post IR (46). The same study revealed that EPO stimulated profibrotic transforming growth factor β, oxidative stress and phosphorylation of ERK in IR kidneys, and also promoted epithelial-to-mesenchymal transition and activated fibroblasts under oxidative stress *in vitro*. Through overexpression of EPOR in tubules, evidence also demonstrated that higher EPO/EPOR signaling caused fibrosis in the IR-injured kidney at 14 days post IR by suppressing basal autophagy activity and upregulating apoptotic events through modifying microtubule-associated protein 1A/1B-light chain 3 and active caspase-3 expression, respectively (47). This evidence indicates that the homodimer (EPOR)_2_ could contribute to the fibrotic development of IR kidneys, as the heterodimer EPOR/βcR was demonstrated to have an anti-fibrotic role in the kidney after IR through its specific ligands such as CHBP (45). Thus, an advantage of EPO derivatives is that they only specifically bind to EPOR/βcR, avoiding the side effects of EPO caused by not only erythropoiesis but also pro-fibrosis. Understanding the expression, regulation, and function of (EPOR)_2_ and EPOR/βcR under different diseases would be beneficial to increase the effectiveness of their respective ligand drugs, reduce side effects, and avoid invalid use.

## Physical Interaction Between EPOR and βCR

The protective role of EPO revealed in different organs indicates that a variety of non-hematopoietic tissues have the receptor of EPO. EPOR, a member of the type I cytokine receptor family, is present on the surface of erythroid progenitor cells at a high level, and assembly of the homodimer (EPOR)_2_ mediates erythropoiesis upon receiving signals from EPO (49). During the developmental stages of embryos, EPOR was found broadly expressed in many organs and cells, such as the kidney, brain, and heart (50–52). It appeared predominantly in the kidney after the first two trimesters. Knockout of EPOR in a mouse fetus resulted in death of the mouse, which may be partially due to erythropoiesis and the development of organs including the kidney (53). Thus, the signaling of EPO/EPOR is crucial in the normal development of mouse fetuses, showing its potentially broader roles apart from erythropoiesis. However, EPOR expression was gradually decreased in the kidney cortex and became stable until the maturation of the kidney postnatally (54). In adult rats, EPOR expression in kidneys remains very low or absent under normal conditions, mainly in TECs (55). This expressional change of EPOR indicates its multiple functions that may be involved in the different periods of life.

Alike EPOR, βcR also belongs to the type I cytokine receptor family. βcR is a shared common β chain with a receptor of interleukin (IL)-3, IL-5, and granulocyte-macrophage colony stimulating factor (GMCSF), which binds to a corresponding α chain (56–58). βcR mainly locates on the surface of hematopoietic cells and exerts immune regulation (59). In 1995, βcR was found to be responsive to EPO as the tyrosine phosphorylation of βcR was observed upon EPO treatment to cultured cells (60). Paul and his colleagues (61) transfected B lymphocytes with vectors expressing both EPOR and βcR genes and then obtained their heterologous expression in these cells. The two receptors associated with each other as EPOR/βcR were demonstrated in transfected cells by the detection of co-immunoprecipitation. In 2004, Brines and his colleagues demonstrated that EPOR and βcR were physically linked *via* cysteine residues (24), which might be already assembled in the absence of the EPO molecule (61). Co-expression of EPOR and βcR has been detected in various organs, such as the kidney, heart, and nervous system (62, 63).

## Signaling Pathways Mediated By EPOR/βCR

**Figure 1:**
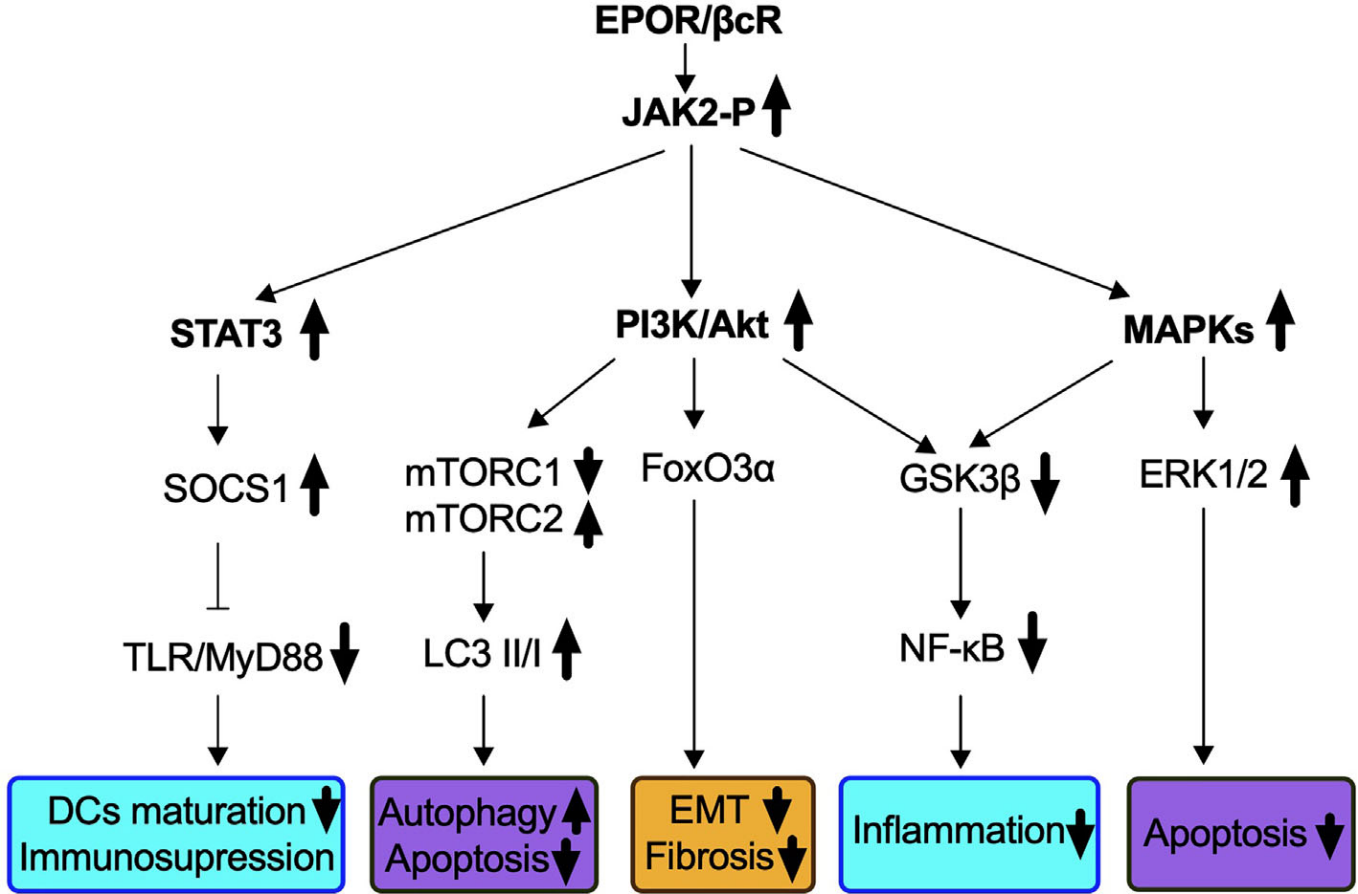
Signaling pathways identified in IR-induced AKI *via* EPOR/βcR activation. EPO-derived tissue protective components such as CEPO, HBSP, and CHBP lead to the phosphorylation of EPOR/βcR-linked JAK2, and subsequently activates several downstream cascades. Biological processes that were influenced through EPOR/βcR signaling included immune response (blue box), cell death (purple box), and kidney repair/preventing renal fibrosis (yellow box).

EPOR and βcR do not contain an intrinsic kinase domain for downstream signaling but could induce Janus kinase 2 (JAK2) auto-phosphorylation by spatial conformational change upon receptor occupancy (60, 64, 65). As EPO signals go through both homodimer and heteromer receptors, molecular pathways specifically associated with EPOR/βcR were demonstrated by non-erythropoietic tissue-protective compounds, primarily HBSP and CEPO (42). Three dominant pathways followed by JAK2 phosphorylation have been identified, which were similar to those used in the hematopoietic system followed by homodimer receptor (EPOR)_2_ binding (**Figure 1**).

Firstly, the phosphatidylinositol 3-kinase (PI3K)/Akt (also known as protein kinase B) pathway was involved through EPOR/βcR in the kidney, liver, and heart (66–70). In a mouse renal IR model, autophagy was induced by CHBP *via* inhibition of mammalian target of rapamycin complex 1 (mTORC1), but activation of mTORC2 led to renoprotective effects (44). Forkhead box O 3α (FoxO3α), a downstream effector of Akt, was activated by CHBP and linked to anti-fibrotic effect in a kidney IR model (45). Nevertheless, upon CEPO treatment, FoxO3α was dephosphorylated and consequently stimulated p27 expression, which was a key factor responsible for the negative regulation of cell cycle and cell proliferation demonstrated in human acute myeloid leukemia cell line UT-7 (71).

The second major molecular pathway involves signal transducer and activator of transcription 3 (STAT3) signaling. Phosphorylation of STAT3 in murine kidneys was found upregulated by IR insult, along with decreased apoptotic events (44). The activation of the STAT3 pathway was reported to occur in a cardiovascular system insulted by IR by the blockade of apoptosis and inflammation (68, 69, 72). In addition, in a rat renal allograft model, JAK2/STAT3 signaling induced the downstream pathway of the suppressor of cytokine signaling 1 expression for inhibiting toll-like receptor-induced dendritic cell maturation, as well as pro-inflammatory cytokine production of these cells (73). Most recently, the same group discovered that the JAK2/STAT3 pathway was also essential to enhance the immunosuppressive capability of myeloid-derived suppressor cells (74).

A third pathway refers to mitogen-activated protein kinases (MAPKs) identified in the heart, liver as well as the kidney *via* EPOR/βcR signaling (67–69). Extracellular signal-regulated kinase (ERK)1/2, a downstream pathway of MAPK, plays an important role in the protection of EPO from IR-induced renal apoptosis (75). Activation of ERK signals also accelerates repair of TECs and inhibits progression of interstitial fibrosis, and its correlation with EPOR/βcR needs investigation (76).

Additionally, both the PI3K and MAPK pathways inhibit glycogen synthase kinase 3β (GSK3β), which leads to stabilization of the mitochondrion, reduces oxidative stress damage, and subsequently inhibits death signals (72, 77). HBSP enhanced the phosphorylation of GSK3β in the rat kidney post IR and ultimately reduced inflammation by inhibiting nuclear factor-κB (NF-κB) (39). Moreover, an oxidative pathway including NADH-ubiquinone oxidoreductase Fe-S protein 6, alpha-aminoadipic semialdehyde synthase, and ATP-binding cassette subfamily D member 3 was also elicited by EPOR/βcR, leading to the renoprotective effect of CHBP (78).

## Renoprotection Mediated By EPOR/βCR

**Figure 2:**
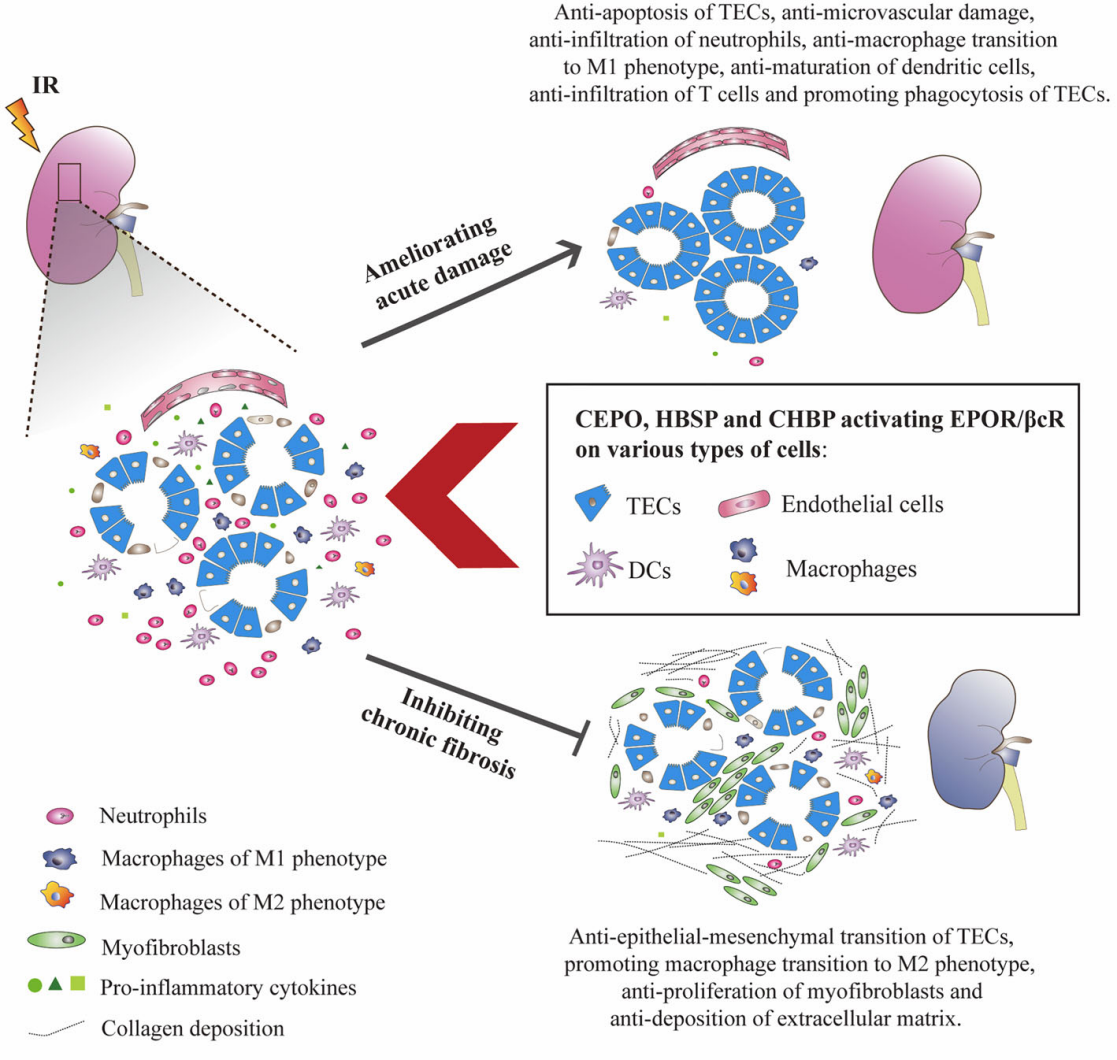
EPO derivatives with tissue protective features benefited IR-injured kidneys at both acute and chronic stages. CEPO, HBSP and CHBP occupied EPOR/βcR on identified cells, reduced the apoptotic death of TECs, and the infiltration, pro-inflammatory transformation and maturation of inflammatory cells at the acute injury stage. The potential role of EPOR/βcR on the phagocytic function of TECs needs further study. These EPO derivatives also promoted the proliferation of TECs and transformation of macrophages to the M2 phenotype (anti-inflammatory) and reversed the proliferation of myofibroblasts and deposition of extracellular matrix proteins including collagen in interstitial areas at the chronic repair stage.

The signaling pathways of EPOR/βcR bring multiple favorable effects on IR-injured kidney, not only against the injury, but also promotes repair. Upon IR insult, kidney cells were stressed with energy exploitation, waste retention, and accumulation of reactive oxygen species, then underwent sublethal, lethal damage, resulting in the release of alarm signals and evoking a sterile inflammatory reaction (7). Although the kidney has the capability to self-renew, maladaptive repair characterized as the deposition of collagen in tubulointerstitial areas cannot be avoided completely (79). As expected, the instant injury at an early stage of IR injury was greatly reversed by EPO and its derivatives through ameliorating kidney apoptosis and inflammatory response. Moreover, early intervention of EPO derivatives seems to turn on the switch of long-lasting biological actions as it can also inhibit chronic renal fibrosis. It was thought that EPOR/βcR mediated the balance of the microenvironment at injury sites, which benefited the recovery of the injured organ. However, the exact mechanism is still not very clear. The cell types in the kidney possessing EPOR/βcR were summarized (**Figure 2**), with detailed discussion below.

### Ameliorating Apoptotic Cell Death

Injury of renal TECs was the most predominant pathological change of renal IR injury. These cells demand a high amount of oxygen and ATP, and thus are susceptible to ischemic insult. Severe stress to TECs could lead the cells to a variety of cell death modes including apoptosis (80). In both rodent and large animal experiments, inhibiting apoptotic pathways demonstrated substantial benefits to the recovery of IR kidneys (81, 82). Anti-apoptotic effect through EPOR/βcR in IR-induced AKI was also evidenced in animal models (3, 39, 83). The underlying mechanism could be manifested by the instant expression of EPOR/βcR on kidney TECs, which helps to construct an image that HBSP directly binds to EPOR/βcR on these cells and transmits protective signals inhibiting apoptosis (11). Evidence also suggested that the anti-apoptotic effect of EPOR/βcR activation would also contribute to the reduced level of oxidative (78) and enhanced autophagy *via* inhibition of mTORC1 and activation of mTORC2 (44), which rescues the TECs from apoptotic cell death. In addition, sustained dysfunction of the endothelium post reperfusion results in the obstruction of capillaries and a local ‘no-reflow’ phenomenon, which could lead to continuous hypoxia and cumulative apoptosis (84). Sautina et al. defined the association of vascular endothelial growth factor receptor 2 with EPOR/βcR to elicit downstream signals in the endothelium and attenuated microvascular damage (85, 86). It is indicated that EPOR/βcR would also mediate the maintenance of microvasculature and reduce further damage to tubules.

### Regulating Immune Response

#### Neutrophils, Macrophages, Dendritic Cells, and T Cells

Inflammatory cells accumulated at the site of damage are usually a double-edged sword. These cells cause further damage at the early stage of AKI, and also initiate the clearance of injured cells and inflammation to limit damage and promote tissue repair. However, sustained inflammatory cell infiltration leads to uncontrolled inflammation in the kidney, further tissue injury, and maladaptive repair (87). Others and our previous work demonstrated that HBSP reduced infiltration of neutrophils and production of pro-inflammatory cytokines such as IL-1, IL-6, and tumor necrosis factor α by inhibiting the NF-κB pathway in rodent renal IR injury models (39, 44). Furthermore, in a large animal model, CHBP protected isolated porcine kidney and restored renal function by reducing interstitial neutrophils and decreasing pro-inflammatory cytokine transcripts 3 h after reperfusion (3). It has been found that EPOR/βcR presented or functioned in several types of immune cells, such as macrophages and dendritic cells (25). HBSP inhibited the transformation of macrophages to the pro-inflammatory M1 phenotype (88) but markedly increased the ratio of the anti-inflammatory M2 phenotype (89), of which the latter supports the transition from tubule injury to tubule repair in IR kidneys (90). In addition, CHBP also inhibited the maturation of dendritic cells through the JAK2/STAT3 signaling pathway (73), suggesting the expression of EPOR/βcR on dendritic cells. This effect of CHBP also suppressed the infiltration of T cells, which would reduce tissue damage caused by excessive immune activation (91). The expressional and functional profile of EPOR/βcR on dendritic cells, antigen-presenting cells, and T cells should be further defined, which may influence the outcome of diseases.

#### Phagocytosis of TECs

Additionally, timely clearance of damaged cells also benefits the injured kidney from secondary inflammatory injury (92). Bangwei Luo et al. reported that apoptotic cell-released sphingosine 1-phosphate activated EPO/EPOR signaling in macrophages, which enhanced dying cell clearance (93). However, whether the phagocytic function of macrophages was enhanced through EPOR homodimer or hetero-receptor EPOR/βcR upon EPO binding remains unclear. Nevertheless, surviving TECs are the main (semi-professional) phagocytes to uptake dead cells in the kidneys subjected to IR injury by expressing kidney injury molecule-1 (KIM-1) (94). KIM-1, a specific molecule expressed by TECs upon injury, recognizes phosphatidylserine on apoptotic cells and transforms TECs to phagocytes (94). The mechanism of EPOR/βcR ameliorating IR injury in kidneys may also relate to the phagocytic function of TECs and facilitate clearance of apoptotic cells. Using a bioinformatics method, STAT3 was identified as an upstream regulator of KIM-1 expression (95), which could also be activated *via* EPOR/βcR. It is suggesting that ligands to EPOR/βcR might trigger signals for TECs turning into phagocytes.

#### Promoting Regeneration/Remodeling

The kidney could recover from IR injury that resulted in substantial loss of TECs (96). Nevertheless, kidney repair is often maladaptive when the injury is severe or mild but frequent (97). The sources of cells that contribute to the replenishment of the population of TECs after injury mainly originate from endogenous surviving TECs (98). Many injury factors, especially long-term hypoxia resulted from sustained loss of peritubular microvessels (99), and disturbance of immune-respondent components such as chronic activation of macrophages of the M1 phenotype (100, 101) have been suggested to contribute to post-ischemic fibrosis. These factors may then induce epigenetic changes in resident myofibroblasts, which result in prolonged fibroblast activation and fibrogenesis (102). Imbalanced regeneration of renal parenchymal cells and repair were characterized by interstitial fibrosis that might be the key determiner for the fate of the injured kidney in terms of its long-term outcome.

The activation of EPOR/βcR was observed to decrease the expression of α-smooth muscle actin (α-SMA), a specific marker for myofibroblasts, and deposition of the extracellular matrix in rodent IR injury and unilateral ureteral obstruction models (44, 83). EPOR/βcR signaling also compromised the pro-fibrotic effect of transforming growth factor-β in cultured TECs by maintaining the level of E-cadherin and lowering vimentin, attenuating their epithelial-mesenchymal transition *in vitro* (3, 45). This action would contribute to the reduced expression of pro-fibrotic FoxO3α on TECs through EPOR/βcR signaling. In addition, CEPO, which specifically recognizes EPOR/βcR, decreased α-SMA expression, a marker of myofibroblasts, in a 14-day rat AKI model induced by unilateral ureteral obstruction (103). These findings suggest that signals after EPOR/βcR activation would block the way of fibrogenesis by modifying the behavior of both parenchymal cells and interstitial cells, however, the exact mechanism needs further investigation. Evidence has shown that EPOR/βcR mediated the proliferation of endothelial cells and neural progenitor cells (26, 104). However, whether EPOR/βcR mediates the dedifferentiation and proliferation of surviving TECs is unknown. Moreover, communication among cells is critical to kidney repair, such as crosstalk between TECs and myofibroblasts, pericytes, or macrophages, which would lead to regeneration of kidney parenchymal cells or the bulk production of the extracellular matrix. Investigation of EPOR/βcR on cell-to-cell communication would not only lead to further understanding of its pro-repair and anti-fibrotic action but also benefit the determination of the pathogenesis of renal IR injury.

## EPOR/βCR as Potential Biomarker

### Regulation and Modulation of EPOR/βcR

On a genomic level, the upstream regulation of EPOR expression has been studied in various studies involving the kidney. Hypoxia inducible factor-1α (HIF-α) is a major transcription factor mediating the cellular response to hypoxia and plays a pivotal role in the resolution of AKI (105). Exogenous HIF-α improved the survival of rat AKI induced by IR insult, as well as increased the downstream effector EPOR, indicating the association between EPOR and HIF-α (106). In addition, overexpression of transmembrane Klotho or administration of secreted Klotho exerted protective effects against IR-induced AKI (107, 108). Hu and colleagues reported that EPOR was a downstream effector of Klotho (55, 109). It was also suggested that tumor necrotic factor α and its receptor were required for the upregulation of EPOR and the therapeutic effects of EPO (110, 111). Furthermore, Youn-Soo Lee et al. found that deficiency of the Von Hippel-Lindau gene resulted in the development of tumors in the kidney, characterized by consistent upregulation of EPO and EPOR in renal carcinoma (112). Thus, it indicated that Von Hippel-Lindau might be an upstream inhibitor of EPO or EPOR, of which the latter genes are responsible for the proliferation of immature mesenchymal cells (113). Besides, EPO induces the ubiquitination and internalization of EPOR *via* clathrin-mediated endocytosis, following by the degradation of its intracellular part by the proteasome, preventing further signal transduction (114, 115). Nevertheless, the regulation of βcR expression in kidney parenchymal cells at the genomic level remains unreported, for which studies might be limited by the lack of specific ligands for (βcR)_2_. The potential effect of upstream genes on EPOR regulation and EPOR/βcR formation for enhancing protection needs to be further explored.

Both EPOR and βcR belong to the type I cytokine superfamily, of which one family member usually creates a complex with the other in the same family as stimulated by the ligand. In addition, EPOR could be translocated from the nucleus to the cytoplasm and cellular membrane under hypoxia (116). In addition, it was claimed that EPOR could be recruited into membrane lipid raft fractions within 1 min post the exposure of the growth factor, reaching a peak at 10 min (117). There is also evidence regarding the βcR migration to lipid rafts under stimulation of the ligand GMCSF in hematopoietic cells (118). Similarly, the altered cell surface expression of βcR in fibroblasts and macrophages that immediately occurred under stress indicated the swift translocation of the receptor (119). It was assumed that homodimer (EPOR)_2_ and (βcR)_2_ translocated from the cytoplasm to the plasma membrane quickly upon stimulation and assembled a heteroreceptor at the molecular level. Additionally, the regulation of heteroreceptor EPOR/βcR expression has been also shown by its ligand treatment in different disease models. CEPO promoted the complex association of EPOR and βcR on myocardium at 4 h post-reperfusion in a mouse myocardial IR injury model (70). However, the level of EPOR/βcR was decreased by HBSP in a 48-h mouse renal IR model, which may contribute to the decreased degree of kidney injury (11). These regulatory observations indicated potential differences in the underlying mechanisms of EPOR/βcR expression.

### Potential Diagnostic Biomarker

Up to date, the diagnosis for AKI in the clinic mainly relies on the change of serum creatinine level and/or urine output (120). However, the functional alteration of the kidney always results from massive structural disorder, so the supreme timing for targeted therapeutics is often missed, which is crucial for the long-term outcome. Minor damage in the kidney might result in the poor prognosis without timely diagnosis and intervention (97). An ideal biomarker is one that can diagnose AKI at the early stage, monitor the development of the injury, direct the therapeutic interventions, and predict the prognosis of disease (33). The instant expression of EPOR/βcR in the kidney upon renal stress or insult suggested that EPOR, βcR, and/or EPOR/βcR might be a potential early biomarker(s) for IR-induced AKI. However, kidney biopsy is not a routine examination for AKI patients. Dropped off damage parenchymal cells and/or apoptotic inflammatory cells to the tubular lumen provided an opportunity for detecting these receptors in the urine, which might also reflect the level of these receptors in the kidney and the occurrence of renal injury. However, there is no available report regarding EPOR/βcR in plasma and urine so far, which may be due to the challenge of EPOR/βcR detection. Nevertheless, EPOR was found as a soluble protein in plasma that corresponds to the extracellular domain of EPOR on the cellular membrane. Biologically, the soluble EPOR exists in human plasma to bind excessive EPO and maintains the latter at a constant level (121). It has been reported that the level of soluble EPOR may contribute to erythropoietin resistance in end stage renal disease, and that its production may be mediated by pro-inflammatory cytokines such as TNF-α and IL-6 (122), indicating soluble EPOR as a potential biomarker of cell injury. However, the regulation of soluble EPOR in the plasma of animals or humans with IR-injured kidneys remains unclear. Understanding the expression and regulation of EPOR in different forms, as well as EPOR/βcR could benefit its potential application in AKI diagnosis and progression.

Therefore, exploration of the dynamic expressional profile of EPOR and potentially EPOR/βcR in the serum and urine post renal IR at different stages of injury would provide essential evidence to assess the diagnostic potential of these receptors. The association between the expression of these receptors and the development of renal IR injury might give information on the monitoring of disease. Early intervention using EPO derivatives that specifically activate EPOR/βcR will be beneficial for not only the short-term but also long-term outcome of IR-induced AKI. The dynamic change of EPOR/βcR in the kidney post insults such as IR at the initiation and the development of injury, as well as repair stage, might direct the application of specific ligand drugs.

## Challenges and Opportunities

Apart from the availability of the clinical specimens, the sensitivity and specificity of detection for the level of EPO and its receptors including EPOR, βcR, and EPOR/βcR in liquid specimens should be optimized. Especially for the heterocomplex, a co-immunoprecipitation assay needs a large sample with adequate expression. Whether EPOR or βcR protein, as well as the mRNA level of these receptors, could represent the level of heteroreceptor remains unclear and requires further study. Additionally, the complexity of disease may compromise the sensitivity of detection as various diseases may increase the expression of this protective receptor especially in patients with problems in other organs except the kidney or possessing complex kidney problems. Besides, there is also crosstalk between the kidney and other organs such as the brain, liver, and lung post renal IR (123). The level of EPOR, βcR, or EPOR/βcR in urine might not reflect the exact injury in the kidney, making the assessment of renal injury difficult. However, the functions, underlining mechanisms, and diagnostic potential regarding EPOR/βcR in AKI would also be applicable to other organs with acute injury. There are clinical trials using HBSP (also named ARA290) on neural systems (NCT02070783, NCT02039687) and the endocrine system (NCT01933529) indicating that HBSP possesses a wide range and great potential for clinical applications in different organ systems. EPOR/βcR has been found widely expressed in many kinds of cells including progenitor cells. The potential side effect of *in vivo* administration of EPOR/βcR ligands such as HBSP/CHBP in the clinic shall consider the potential that the repair signal of the receptor might lead to excessive proliferation of progenitor cells, resulting in tumorigenesis or fibrosis. In addition, EPOR/βcR might also be a natural guider for specific cell target drug delivery such as small interfering RNA conjugated with its ligand HBSP or CHBP, as TECs are most vulnerable to IR injury and highly expressed EPOR/βcR.

## Conclusion

The timely and spatial expression of EPOR/βcR in the injured kidney upon IR suggests its potential for early diagnosis and monitoring progression, as well as cell target drug delivering such as siRNA conjugated to its ligand. The key facts and messages are summarized as follows:

1. Hypoxia and inflammatory cytokines stimulate the swift translocation of EPOR and βcR to the cellular membrane and assemble the heterodimer.
2. EPO-derived peptide HBSP or CHBP only activates EPOR/βcR, possessing great renoprotection against IR injury. The metabolic profile and potential side effect of EPO derivatives should be further investigated before their clinical applications.
3. The signaling pathways of PI3K/Akt, STAT3, and MAPK were involved in the renoprotective effects of EPOR/βcR such as anti-apoptosis of TECs and endothelial cells and anti-inflammation at the acute stage, promoting proliferation of parenchymal cells and anti-fibrosis at the chronic stage.
4. Highly expressed EPOR/βcR in damaged cells provides a great opportunity for cell target delivery of drugs that was conjugated with its ligand such as siRNA conjugated with HBSP to precisely treat IR-induced AKI, promote repair/remodeling, and prevent its chronic progression to fibrosis.

## Author Contributions

BY and YW contributed to the conception and design of the article. The review was written by YW and revised by BY. Both authors approved the final version of the manuscript.

## Funding

This work was supported by the grants from the National Natural Science Foundation of China (81873622) and the Leicester Kidney Care Appeal (2018); the Research and Innovation Department, the University Hospitals of Leicester NHS Trust, and the University of Leicester.

## Conflict of Interest

The authors declare that the research was conducted in the absence of any commercial or financial relationships that could be construed as a potential conflict of interest.

The reviewer CS declared a shared affiliation with one of the authors BY to the handling editor at the time of review.
